# A community-based health education programme for bio-environmental control of malaria through folk theatre (*Kalajatha*) in rural India

**DOI:** 10.1186/1475-2875-5-123

**Published:** 2006-12-15

**Authors:** Susanta K Ghosh, Rajan R Patil, Satyanarayan Tiwari, Aditya P Dash

**Affiliations:** 1 National Institute of Malaria Research (ICMR), Epidemic Diseases Hospital, Old Madras Road, Bangalore – 560 038, India; 2 Community Health Cell, Kormangala, Bangalore – 560 034, India; 3 National Institute of Malaria Research (ICMR), 22-Sham Nath Marg, Delhi – 110 054, India; 4 Integrated Disease Surveillance Programme [WHO, UNDP], UN House-II, 256, Forest Park, Bhubaneswar, Orissa – 751009, India

## Abstract

**Background:**

Health education is an important component in disease control programme. *Kalajatha *is a popular, traditional art form of folk theatre depicting various life processes of a local socio-cultural setting. It is an effective medium of mass communication in the Indian sub-continent especially in rural areas. Using this medium, an operational feasibility health education programme was carried out for malaria control.

**Methods:**

In December 2001, the *Kalajatha *events were performed in the evening hours for two weeks in a malaria-affected district in Karnataka State, south India. Thirty local artists including ten governmental and non-governmental organizations actively participated. Impact of this programme was assessed after two months on exposed *vs. *non-exposed respondents.

**Results:**

The exposed respondents had significant increase in knowledge and change in attitude about malaria and its control strategies, especially on bio-environmental measures (p < 0.001). They could easily associate clean water with anopheline breeding and the role of larvivorous fish in malaria control. In 2002, the local community actively co-operated and participated in releasing larvivorous fish, which subsequently resulted in a noteworthy reduction of malaria cases. Immediate behavioural changes, especially maintenance of general sanitation and hygiene did not improve as much as expected.

**Conclusion:**

This study was carried out under the primary health care system involving the local community and various potential partners. *Kalajatha *conveyed the important messages on malaria control and prevention to the rural community. Similar methods of communication in the health education programme should be intensified with suitable modifications to reach all sectors, if malaria needs to be controlled.

## Background

### The threat of malaria

Malaria is a major public health threat to the developing world, indirectly affecting the economic development. Nearly 40% of the world's population is at risk and 80% of the burden exists in sub-Saharan Africa. Almost all the remaining cases exist in tropical and subtropical Asia, Latin America and Melanesia [1]. In India, less than two million cases with few hundred deaths are recorded every year [2,3], but the estimated number is 15 million with about 19,500 deaths [4]. Karnataka state, south India contributes approximately 7–10% of India's annual malaria burden [5].

### The need for health education in malaria control programme

Unlike HIV/AIDS, sufficient emphasis has not been given to health education in malaria control programmes. This has resulted in poor community acceptance and involvement in the various control strategies undertaken [6]. WHO under the Roll Back Malaria (RBM) initiative recognizes the need for community participation and inter-sectoral co-ordination involving various like-minded partners for effective programme implementation [7]. Community being the stakeholder, it is essential that information about diseases and their control methodologies should be made available to them [8].

There is no standard format for delivering health education messages. Many conventional methods such as posters, pamphlets, hoardings and electronic media, have limited effects on the rural community due to their low literacy rate. In such situation, *Kalajatha *(folk theatre) as a medium of mass communication has been experimented to assist the malaria control programme.

### Background to the study

Each year, nearly 50% of malaria cases in Karnataka were reported from the districts of Tumkur, Hassan, Chickmagalur and Chitradurga [5]. *Anopheles culicifacies *is the primary malaria vector, which breeds mainly in wells, streams and irrigation ponds [9]. The traditional method of malaria control using indoor residual spraying with insecticides even with synthetic pyrethroids did not produce the expected result. A kind of frustration was prevailing in the local community. In a silk producing area of Kolar district, local farmers were reluctant to the use of DDT spaying because of the perceived deleterious effect on silk worms. In this area, bio-environmental control of malaria especially larvivorous fish is very effective in controlling *An. culicifacies *[10,11]. Tumkur was one of the five districts in India selected for situational analysis under RBM. The expert committee recommended the need for health education in malaria control [12]. Based on this, the present programme was initiated with the following objectives:

i. to assess the operational feasibility and communication efficacy of *Kalajatha *in health education programme for bio-environmental control of malaria.

ii. inter-sectoral co-ordination and involvement of all potential partners in health education.

## Methods

### Population and the area served

The *Kalajatha *programme was organized in Primary Health Centre (PHC) Mathigatta under Chikkanayakanahalli taluka, Tumkur district which was badly affected by malaria [5]. This *taluka *(secondary revenue division) has 264 villages covering an area of 112,998 hectares with a population of 215063 in 2001. The villages are administered by 28 *Gram Panchayats *(village elected representation). Health care services are provided through eight PHCs. PHC Mathigatta has 58 villages with a population of 28253. The literacy rate was 63%. The male to female sex ratio was 0.97. Infant mortality rate was 50 per 1,000 live births. The birth rate is double the death rate. Agriculture, horticulture, and animal husbandry are the main economic activities, which engage almost 80% of the workforce. Coconut is the main cash crop. Agriculture provides only seasonal employment and the returns are low. Non-agricultural economic activities are poorly developed. The annual rainfall ranges from 600 to 800 mm while temperature is between 13°C and 39°C. The peak malaria transmission period is in the months May and June.

### The partners and planning

The National Institute of Malaria Research (NIMR) and Community Health Cell (CHC), Bangalore, jointly initiated the programme. An inter-sectoral co-ordination committee was formed involving ten governmental and non-governmental organisations for smooth functioning. The district health committee headed by the District Commissioner approved the proposal of the *Kalajatha *programme. NIMR and CHC, Departments of Health, Education, Child and Women's Welfare, Rural Development and *Panchayat *Raj, Tumkur Science Forum, local political and religious leaders actively participated in this programme.

**Figure 1:**
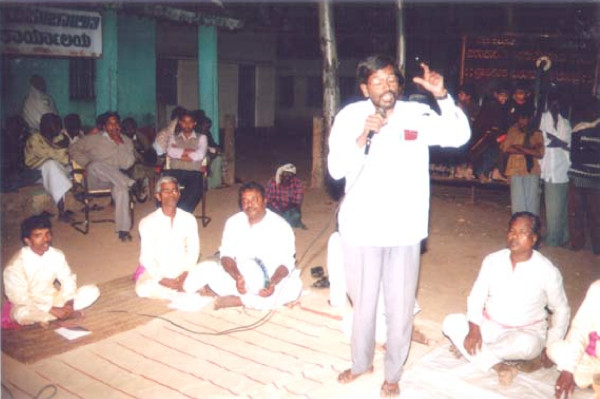
Rehearsal of the *Kalajatha *programme in the evening at the *Kuppur Mutt *(religious trust). A trainer was directing the artists.

Thirty local artists (15 males and 15 females) from different occupational background were selected. A local scriptwriter wrote 8 songs, two dramas and 4 *rupakas *(musical dramas). The scripts were based on various aspects of malaria namely signs and symptoms of sickness, treatment, health facilities, processes of transmission, role of anopheles mosquitoes and names of the malaria vectors, breeding grounds of mosquitoes especially the vectors, its control strategies focusing especially on larvivorous fish (*Poecilia reticulata *and *Gambusia affinis*) and environmental management. Other control strategies like insecticide-treated nets, adopting measures for maintaining general hygiene, keeping cleanliness in and around houses, and the role of the community were also included in the script. These were then translated into skits using local dialects, musical styles and theatre traditions. In the beginning, the artists underwent orientation training on the entire processes. Two troupes consisting of 15 artists each were formed. Before the actual performances, they rehearsed the events in the evening for two weeks in a religious trust of *Kuppur Mutt *(Figure 1). Each troupe was equipped with a set of musical instruments and a performing wardrobe.

### The *Kalajatha *events

**Figure 2:**
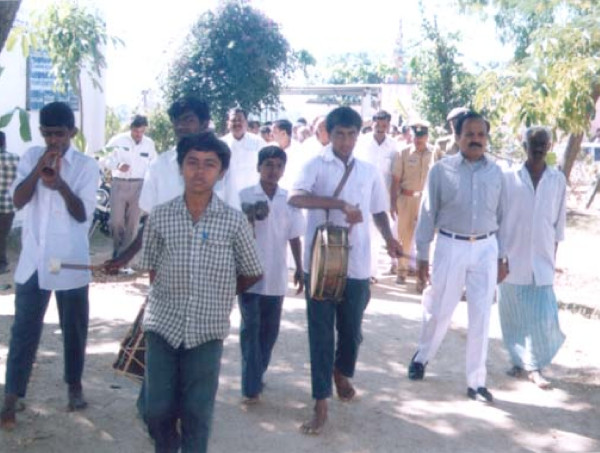
Public awareness campaign for the *Kalajatha *programme. Local high school children, teachers and *Gram Panchayat *members took part in the campaign.

**Figure 3:**
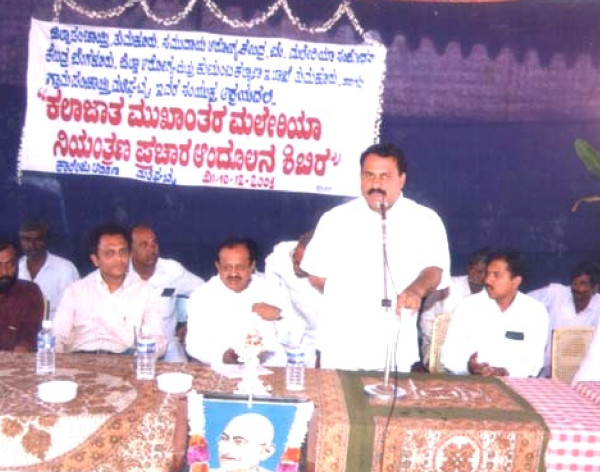
Inauguration of the *Kalajatha *programme, December 2001. The Minister-in-Charge of Tumkur district, local *Gram Panchayat *members, Head, *Kuppur Mutt*, district health officials, members from NIMR, CHC, Bangalore and others were present.

**Figure 4:**
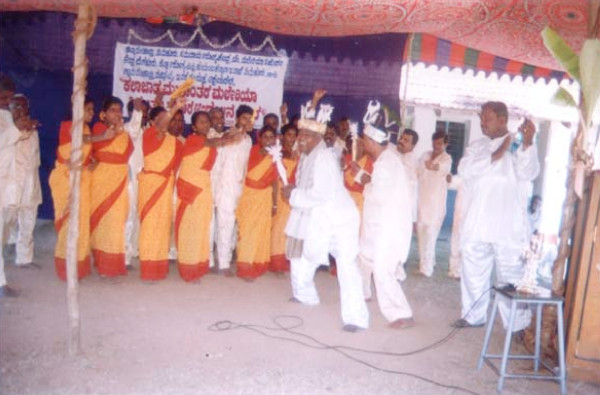
A glimpse of the *Kalajatha *programme performed by the artists. The artists are presenting the various sings and symptoms of malaria.

**Figure 5:**
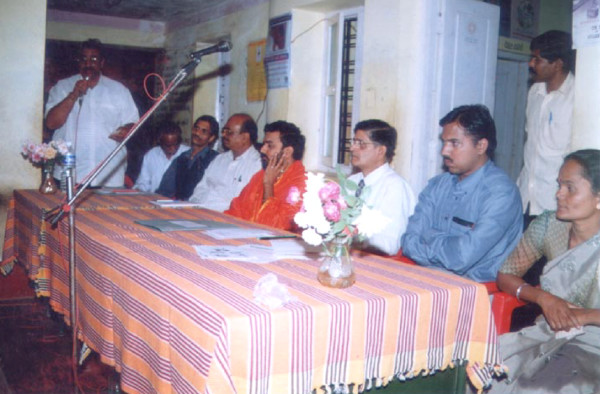
Sharing the experience of the *Kalajatha *programme in the valedictory function. The Director, Health and Family Welfare Services; Head, *Kuppur Mutt*, members from NIMR, CHC and local *Gram Panchayat *members and other local health officials participated in the function.

The *Kalajatha *events were performed in December 2001. One week before the events, wide publicity was given through the local village administration (*Gram Panchayat*) and the community consent was obtained from the village headmen or *Panchayat *presidents (Figure 2). The Minister-in-Charge of the district and the local elected legislative assembly members inaugurated the programme (Figure 3). The events were performed in the evenings so that maximum number of people could witness. Every day, each troupe visited two villages and spent two hours in each village. Villagers voluntarily attended the programmes (Figure 4). Local health officials and *Gram Panchayats *provided all the necessary logistics and hospitality. A valedictory function was held at the end, which was presided over by the Director of Health Services, Karnataka (Figure 5). Local media covered the events and helped in spreading the key messages.

### Impact assessment

Two months after the events, impact was assessed in five villages of PHC Mathigatta (exposed) and in another five villages of PHC Dasaudi (non-exposed) chosen at random. Semi-structured interviews based on eight questionnaires were conducted with individual households. In each village, households were selected randomly and considered as one unit. All the individuals in the house present at that time were interviewed. Children below eight years were excluded. Responses between the exposed and the non-exposed respondents were analyzed following Fisher Exact and χ^2 ^tests, wherever applicable.

## Results

Data on the Kalajatha responses are shown in Table 1. Of the total 87 households interviewed 48 were from PHC Mathigatta (exposed) and 39 from PHC Dasaudi (non-exposed). In the exposed households, 152 (23 children and 129 adults) and in the non-exposed households 137 (17 children and 120 adults) respondents respectively were interviewed. The exposed respondents significantly gained new knowledge about malaria, its symptoms, transmission and control methodologies (*P *< 0.001). They could easily associate clean water with anopheline breeding and recall the names and the role of larvivorous fish guppy (*Poecilia reticulata*) and *Gambusia affinis *in control of malaria vectors. However, immediate behavioural changes especially in maintenance of general hygiene were not observed. The budget breakdown of the events is summarized in Table 2. The per capita cost for conducting the programme was INR 3.0 (US $ 0.064; 1 US $ = 47 INR).

**Table 1:** Responses of the *Kalajatha *events performed in December 2001

		**Respondents**		
			
**S. No**	**Questionnaires**	**Exposed**	**Non-exposed**	**P**
1	Any new learning	17 children and102 adults responded that they had learnt new information about malaria	None responded correctly	< 0.001^a^
2	Signs and symptoms of malaria	6 children and 93 adults could describe the three stages of malaria; chill, fever and sweat	None could tell correctly	< 0.001^a^
3	Knowledge of malaria transmission	9 children and 57 adults specified correctly	Only 4 school children	< 0.001^b^
4	Name of the malaria vectors	11 children and 61 adults. Children clearly specified female Anopheles mosquito	Only 4 school children	< 0.001^b^
5	Breeding grounds of malaria vectors	19 children and 102 adults clearly specified clear water sources	3 children and 10 adults specified clear water	< 0.001^b^
6	Larvivorous fish in malaria control	19 children and137 adults clearly specified	Only 13 adults specified	< 0.001^b^
7	Names of larvivorous fish	8 children and 18 adults correctly responded	None responded	< 0.001^a^
8	Any physical improvement/changes after the events	All responded positively to change in their attitude towards cleanliness and hygiene. However, no change in practice was observed	Negative response	

^a ^Fisher Exact test; ^b ^Chi-square test; % Effectiveness could not be found due to some responses were 0 (none) in the non-exposed respondents.

**Table 2:** Budget breakdown of the *Kalajatha *programme

**Item**
Grant provided by the State Health Department, Government of Karnataka towards honorarium for 30 artists; local transport from their houses to the PHC head quarter; wardrobes; event management and incidental expenditures for two organisers from Community Health Cell
Approximate amount received in kind:
Kuppur Mutt for in-house one-week training of the artists
Taluka Health Office for providing transport facility from PHC to the respective villages for 15 days
*Gram Panchayat*s for providing refreshments
National Institute of Malaria Research, Bangalore
Total amount spent to cover 58 villages (population 28253) was INR 85000.00 Per capita cost was INR 3.0 (US$ 0.064); 1 US$ = INR 47.

## Discussion

There are many forms of theatres for delivering health messages. Street theatre, folk theatre forum theatres etc. are being used in many countries. In the Indian sub-continent *Kalajatha *is a very lively and highly powerful traditional art of dance and drama (folk theatre) which delivers key messages of the life processes in local dialects and cultural settings. This is slightly different from street theatre. Street theatre is utilized for mobilizing people to participate in controlling tuberculosis, HIV/AIDS, polio, diarrhoeal diseases and also malaria [13-16]. Puppet shows and street theatre is being used extensively in HIV/AIDS control programme [17,18]. In Africa and in North America, in both rural and urban settings, forum theatre is an effective means of health promotion. Projects on women's health, care for patients with mental disorders, and AIDS prevention show the usefulness of this medium for community action programmes [19]. Theatre was used for mobilizing and sensitizing the community for tsetse control in Uganda [20]. In a cross-sectional study, an impact of IEC campaign for tuberculosis and health seeking behaviour was assessed in Delhi and was used as programme performance indicator [21].

Attempts were made to explore this strong medium for bio-environmental control of malaria under the primary health care system. The performances were very lively and motivating and many spectators even offered to act along with the actors. In some events many had reacted and also agitated for not providing the proper treatment and correct information to the community earlier. The biggest information delivered to the community was that Anopheles and Aedes mosquitoes breed in clear water as against the general belief of polluted water where Culex mosquitoes generally breed. Use of biocontrol agents, source reduction of opportunistic breeding of vector mosquitoes, treatment, health education, environmental management, maintenance of cleanliness and personal hygiene are important components of bio-environmental control strategy. This method is very effective in Indian situations [22]. Besides this, various other methods of malaria control including insecticide treated nets were also incorporated in the messages, but the focus was on larvivorous fishes since they are, at the moment, the main intervention in malaria control in the area.

The present study set an example of inter-sectoral co-operation between various heterogeneous groups. Apart from the impact, the process was itself a model of governmental and non-governmental partnership which was timely especially when the government is seeking examples of public-private partnership in health education activities. The education department deputed five teachers while the Child and Women's Welfare Department deputed ten *Anganwadi *(female resident staff) workers for one month. Fifteen members from the local community, with various occupational backgrounds ranging from carpenter to barber, and having artistic acting and singing talent came together as a team. The Government of Karnataka through the Department of health partially funded the programme. Politicians and ministers played their role by accepting the invitation to inaugurate the programme thereby providing wider visibility to the health education programme. Religious leaders contributed by offering free accommodation and hospitality for the period of one month as a token of solidarity in the fight against malaria. The press and radio helped in wider dissemination of health education messages and analyzing the malaria situation of the district. Female artists were involved in the team, which resulted in good responses from the women community. Currently, all the developmental programmes including health are directly executed by the *Panchayat Raj Institution*. The local *Gram Panchayat *members provided maximum support to this programme. Subsequently these members played a major role in disseminating the messages and generated awareness in the entire area. In the following year (2002), the community co-operated actively in a WHO funded project in releasing larvivorous fish for malaria control. The mid-term report revealed that in Chikkanayakanahalli taluka malaria cases have declined from 10,136 in 2001 to 66 (up to September 2006) [23].

The present study was aimed to sensitize and mobilize and its impact on the community using folk theatre to control malaria especially on bio-environmental measures for which no comparable baseline data were available. The data between the exposed and non-exposed respondents indicated that there was no perceived information on the present campaign. In rural areas many festivals and socio-cultural programmes are performed that may have some counter effects on such events. Such issues were taken into consideration while organizing the *Kalajatha *events.

## Conclusion

Health education aims at behavioural changes in individuals and the community. *Kalajatha *was found to be a very effective medium in promoting health education and possibly behavioural changes to the rural community. The immediate behavioural changes especially on maintenance of general hygiene was not observed. However, the first essential step towards achieving behaviour change communication in the community was achieved by providing correct and scientific information on malaria control and prevention through the innovative and traditional medium that the rural community best identified. Implementation of control measures by the authorities would enhance the community's acceptance and bring about major behavioural changes so as to avoid mosquito borne diseases [24]. Efforts were made to convey the correct messages to the community, because wrong messages may have disastrous after-effects. Many still believe two kinds of environmental modifications which are effective against malaria and are unfortunately frequently included in health education posters as anti-malaria measures. These are (a) cutting grass and bush clearance which was shown to be completely ineffective [25]; (b) clearing of garbage to prevent rainwater accumulation that supports breeding of *Aedes *mosquitoes which need to be controlled for dengue outbreaks, but these mosquitoes do not transmit malaria.

Webber [26] has rightly advocated 'a multiplicity of simple methods, carried out by many people who are likely to be more successful in the long term than more complex methods. It will be the community who will finally control malaria, but health authorities must advise and assist them in the ways of achieving this'. In the study area biocontrol of malaria programme is being maintained routinely. People still refer to the *Kalajatha *events and the messages delivered to them earlier. Thus this made indelible marks in the people's mind for a long period. This programme is now utilized for other diseases also. A detailed account has been described in Table 3 for carrying out a *Kalajatha *programme with suitable modifications. Based on the results described in the present paper, the State Health Department is conducting such events for the prevention of HIV/AIDS.

**Table 3:** General steps, in chronological order for conducting a *Kalajatha *programme

**Target: **Local community suffering from a specific disease for which they can contribute in the control programme.
**Partners and planning: **Select the problematic area. Identify the related partners. Form a co-ordination committee involving all potential partners. Arrange funding for the programme. Identify the artists. Conduct the programme in an appropriate season and time. Give wide publicity and seek political and religious support. Rehearse the programme.
**Content: **Compose music and drama based on the local dialects and tradition carrying the key messages of the disease and its control methodologies. Emphasis should be given on their specific role in the control programme.
**Logistics: **Materials for event management e.g. wardrobe, light and sound systems, refreshments, honorarium and transport etc. for the artists should be made available in time.
**Precautions: **Prior consent of the community should be obtained. Other programmes should not coincide in the same area. An orientation workshop is necessary for the collaborating partners before launching the programme. Co-ordination should be maintained at all levels and time.

## Authors' contributions

SKG and RRP conceived and arranged the entire programme. SNT assisted in conducting the events. APD reviewed and edited the paper. All authors helped write, read and approved the final manuscript.
